# Endoscopic ultrasound-directed transgastric ERCP for disconnected pancreatic duct syndrome: finding the edge to bridge the gap

**DOI:** 10.1055/a-2266-1860

**Published:** 2024-04-23

**Authors:** Mark Henrik Bonnichsen, Prima Bianca Gaffud Chua, Clement Chun-Ho Wu, Ravishankar Asokkumar, Christopher J. Khor, Damien M.Y. Tan

**Affiliations:** 1 37581 Gastroenterology, Singapore General Hospital, Singapore, Singapore; 2 34378 Gastroenterology, Liverpool Hospital, Liverpool, Australia; 3 37581 Gastroenterology, Singapore General Hospital, Singapore, Singapore; 4 470757 Cardinal Santos Medical Center, San Juan, Philippines; 5 Department of Gastroenterology and Hepatology, Singapore General Hospital, Singapore, Singapore; 6 121579 Duke-NUS Graduate Medical School, Singapore, Singapore; 7 37581 Gastroenterology and Hepatology, Singapore General Hospital, Singapore, Singapore; 8 121579 Duke-NUS Graduate Medical School, Singapore, Singapore; 9 121579 Duke-NUS Graduate Medical School, Singapore, Singapore; 10 37581 Dept. of Gastroenterology & Hepatology, Singapore General Hospital, Singapore, Singapore; 11 121579 Duke-NUS Graduate Medical School, Singapore, Singapore; 12 37581 Department Gastroenterology and Hepatology, Singapore General Hospital, Singapore, Singapore

Altered anatomy from Roux-en-Y gastric bypass makes access to the papilla difficult. Solutions such as balloon-assisted and laparoscopy-assisted endoscopic retrograde cholangiopancreatography (ERCP) are complicated by sub-optimal technical success and adverse event rates 1 . EUS-directed transgastric ERCP (EDGE) is a promising solution with high technical and clinical success rates 2 and provides ease of access if multiple procedures are required.

A 60-year-old woman with a previous Roux-en-Y gastric bypass for obesity 8 years before admission presented with severe necrotizing acute pancreatitis. She developed infected pancreatic fluid collections and septic shock secondary to disconnected pancreatic duct syndrome. She underwent multiple percutaneous drainages and laparotomies with washouts over a period of 3 months. She was deemed unsuitable for a distal pancreatectomy. Given the Roux-en-Y anatomy, she was offered EDGE to bridge the disconnected duct.

A linear echoendoscope (EG38-J10UT; Pentax Medical, Tokyo, Japan) was advanced to the Roux anastomosis ( Video 1 ). Assessment at the 49-cm mark from the incisors in the remnant stomach pouch showed a collapsed structure and 19G needle was used to distend this structure to reveal the excluded stomach on fluoroscopy. Direct puncture was performed into the excluded stomach, and a gastro-gastric anastomosis was created using a 20×10-mm lumen-apposing stent system (Axios; Boston Scientific, Marlborough, Massachusetts, USA). We dilated the stent further using a 20-mm balloon dilator. To facilitate successful ERCP and prevent stent migration during the procedure, we sutured the proximal stent to the bowel wall at two sites using the OverStitch device (Apollo Endosurgery, Austin, Texas, USA). We advanced a duodenoscope (ED34-i10T2; Pentax Medical) through the stent, and the pancreatogram showed partial disruption of the duct at the tail of the pancreas leading into a collection ( Fig. 1 **a** ). We performed a pancreatic sphincterotomy, and our attempts to pass a wire across the disconnected duct were unsuccessful ( Fig. 1 **b** ). We then deployed a 5Fr×12-cm single-pigtail pancreatic stent with its tip located within the collection.

EUS-directed transgastric ERCP (EDGE) procedure allowing passage of the standard duodenoscope through the lumen-apposing metal stent to facilitate pancreatic duct cannulation and subsequent stenting for disconnected pancreatic duct syndrome.Video 1

**Fig. 1:**
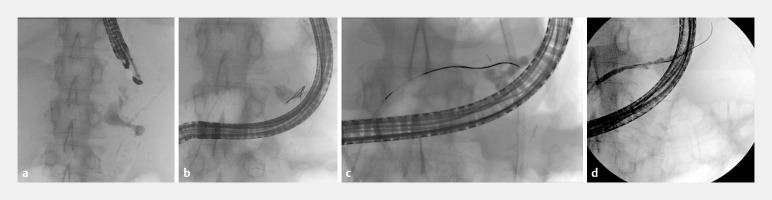
**a** Fluoroscopic images of pancreatic fluid collection with leak and pancreatic duct disruption. **b** Inability to pass the wire into the tail of the pancreatic duct **. c** Pancreatic duct healing after 6 weeks stent dwell time. **d** Complete healing after stent removal at 22 weeks.

A follow-up pancreatogram at 6 weeks showed healing of the disconnected duct, allowing passage of a guidewire ( Fig. 1 **c** ). We deployed a 5Fr×15-cm single-pigtail stent across the disconnected duct. Further imaging showed resolution of the collection and improvement in patient status. The pancreatic stent was removed 22 weeks later, and it showed no further leak ( Fig. 1 **d** ). A progress computed tomography scan shows resolution of the collection.

To our knowledge this is the first case of EDGE for disconnected pancreatic duct syndrome. This syndrome occurs in about 50% of patients with acute necrotizing pancreatitis, and pancreatic stenting has been utilized to bridge the disrupted duct to the viable pancreas upstream 3 . Given the complex clinical presentation, ease of access for multiple procedures and stent insertion permitted healing of the disconnected duct. A particular adverse event of EDGE has been demonstrated to be stent migration 4 . This has been addressed by utilization of a larger lumen-apposing metal stent (LAMS), i.e., 20-mm stent and fixation with endoscopic sutures, which we utilized in this patient. In contrast to established techniques, EDGE provided a solution to a complex clinical problem that required multiple interventions to achieve clinical resolution.

Endoscopy_UCTN_Code_TTT_1AS_2AD

Zitierweise für diesen Artikel

Endoscopy 2024; 56: E41–E42. doi: 10.1055/a-2213-1220

## Notes

Endoscopy E-Videos https://eref.thieme.de/e-videos
*E-Videos* is an open access online section of the journal *Endoscopy* , reporting on interesting cases and new techniques in gastroenterological endoscopy. All papers include a high-quality video and are published with a Creative Commons CC-BY license. Endoscopy E-Videos qualify for HINARI discounts and waivers and eligibility is automatically checked during the submission process. We grant 100% waivers to articles whose corresponding authors are based in Group A countries and 50% waivers to those who are based in Group B countries as classified by Research4Life (see: https://www.research4life.org/access/eligibility/ ). This section has its own submission website at https://mc.manuscriptcentral.com/e-videos .

## References

[1] Schreiner MA Chang L Gluck M Laparoscopy-assisted versus balloon enteroscopy-assisted ERCP in bariatric post-Roux-en-Y gastric bypass patients Gastrointest Endosc 2012 75 748 756 10.1016/j.gie.2011.11.019 22301340

[2] Prakash S Elmunzer BJ Forster EM Endoscopic ultrasound-directed transgastric ERCP (EDGE): a systematic review describing the outcomes, adverse events, and knowledge gaps Endoscopy 2022 54 52 61 10.1055/a-1376-2394 PMC8783372 33506456

[3] Vanek P Urban O Trikudanathan G Disconnected pancreatic duct syndrome in patients with necrotizing pancreatitis Surg Open Sci 2022 11 19 25 36438587 10.1016/j.sopen.2022.10.009 PMC9692037

[4] Shinn B Boortalary T Raijman I Maximizing success in single-session EUS-directed transgastric ERCP: a retrospective cohort study to identify predictive factors of stent migration Gastrointest Endosc 2021 94 727 732 10.1016/j.gie.2021.04.022 33957105

